# Supplementary material for: From Macro- to Microscale: A combined modelling approach for near-surface wind flow on Mars at sub-dune length-scales
Source: PLoS One. 2022 Nov 4;17(11):e0276547. doi: 10.1371/journal.pone.0276547 (PMC9635718; doi:10.1371/journal.pone.0276547)
Supplement: Attachment — Submitted filename: Peer_Review___PLOS_One_August_2022.pdf [file pone.0276547.s001.pdf]

Review of:

"From Macro- to Microscale: A combined modelling approach for near-surface wind flow on Mars at sub-dune length-scales"

The authors present a nested-modeling approach based on a hierarchy of multi-scale solvers to analyze the near-surface wind flow on Mars. A macro-scale general circulation model feeds a meso-scale model which in turn is used to define the boundary conditions for the micro-scale CFD solver. The idea of linking multi-scale models to progress from large scale phenomena to finer scale processes is well-established and the application to study the wind flow on Martian terrain is very interesting. Nonetheless, in its current form, the paper is hard to follow and most of the algorithmic development details are missing. Below, I list a few comments that the authors might want to consider.

1. Pages 9 to 20 were assumed to provide the reader with an understanding of the employed models from NASA-AMES GCM to MRAMS, and finally to the OpenFOAM solver. Instead of that, a very broad and generic description of these models is presented. The reviewer suggests providing more details on the computational setup and a formal definition of the mappings between different levels of solvers.
2. Relevant to my previous comment, the authors refer to some terminologies without prior introduction of those. For example, in Page 12 - Line 257, the authors refer to Grid 1 and Grid 5, but those were not defined. It would be helpful to clearly state the type of grids used in each solver - both in the horizontal and vertical directions. A sketch might be helpful too.
3. The quality of the Figures is not very good, at least in the version that I see as a reviewer. They are blurry and it is almost impossible to read the text inside the figures.
4. In addition to the low quality of embedded figures, the discussions are either too brief or confusing. For example, Figure 1 is supposed to illustrate the workflow of the study, but no discussion of this figure is included, except for a single sentence that cites it. The same comment applies to most of the figures as well.
5. In Figure 9: the authors mention that "The arrow across the central barchan shows the path taken by the mesh independence check". However, it is not clear what this actually means.
6. In Figure 9: the colorbar is only labeled at the 0 and 29.5 marks. It is really hard to interpret the values of the wind speed over the whole domain.
7. The authors apply the CFD model to resolve the wind flow at regions where no data is available. Thus, the only way to validate the results was by making only "qualitative" assessments. However, it is customary to first validate the solver by applying it to regions where data is available so that more quantitative and detailed assessments could be done. After that, the solver can be trusted in regions with less or no data.

In summary, the reviewer concludes covers an interesting topic with a valid modeling approach. However, the manuscript lacks proper description of the computational setup, numerical schemes, and multi-scale coupling algorithms which makes it hard to comprehend the proposed modeling framework. In addition, the low quality of the figures and insufficient/ambiguous discussions hinder drawing insights about the physics of near-surface martian wind flow at the sub-dune length scales.
